# Mechanically Induced Skin Renewal: Evidence‐Based Insights Into Clinical, Structural and Inflammatory Changes Activated by Microneedling, Microdermabrasion and Microvibration

**DOI:** 10.1111/jocd.70908

**Published:** 2026-05-18

**Authors:** Arielle Springer, Elena Helfenbein, Jürgen Blaak

**Affiliations:** ^1^ Dr. Babor GmbH & Co. KG Aachen Germany

**Keywords:** aesthetic treatment, inflammation, microdermabrasion, microneedling, microvibration, skin renewal

## Abstract

**Background:**

Device‐based aesthetic procedures such as microneedling, microdermabrasion and microvibration have gained widespread clinical and institutional use for skin rejuvenation, yet the underlying biological mechanisms and the robustness of clinical evidence remain incompletely established.

**Objective:**

This review synthesizes current clinical, histological and molecular data to evaluate the efficacy, safety and mechanistic pathways engaged by these instrument‐assisted dermatologic interventions.

**Methods:**

A comprehensive literature search of clinical trials, in vivo and ex vivo analyses, and mechanistic studies was conducted to examine outcomes related to clinical skin appearance, inflammatory signaling and protein expression.

**Results:**

This review summarizes clinical, histological, and molecular evidence on these interventions. All modalities influence skin texture, scarring, and epidermal renewal, primarily through transient activation of inflammatory pathways, followed by fibroblast activation and extracellular matrix remodeling. Understanding these pathways can guide treatment selection and optimization, while standardized studies and deeper proteomic analyses are needed to refine personalized approaches.

## 1 Introduction

Skin health and psychological well‐being are becoming increasingly important and depend more on each other in our today's society. Consumers express a desire to feel comfortable in their own skin—and are open to device‐based treatments in skin clinics, cosmetic institutes, and at home to achieve stronger skin improvement and renewal.

In (aesthetic) dermatology, energy‐based treatments have already achieved impressive results in the treatment of scars, skin aging, dyspigmentation, and tissue or vascular changes. While laser therapy (e.g., CO_2_, Er:YAG, diode, pulsed dye laser, Nd:YAG) uses concentrated light of a specific wavelength for the targeted ablation and heating of specific skin layers, thereby activating signal pathways for inflammation and cell regeneration [1, 2], stimulating collagen, skin renewal, providing scar and pigment treatment, and decreasing oxidative stress [3, 4]. Moreover, LED/photobiomodulation works with red, blue, or near‐infrared light across a broad wavelength range to stimulate cell signaling pathways [5, 6], reducing inflammation and oxidative stress [7], and promoting wound healing without tissue damage [8]. Radio frequency (RF) can be applied to the skin externally in a monopolar or bipolar form or fractionated as microneedling RF deep into the skin. The release of electromagnetic energy into dermal layers stimulates collagen and elastin formation and lipolysis [9], while targeted heating in the skin depth between the RF needles builds up collagen [10, 11]. A comparison between fractional radiofrequency needling and bipolar radiofrequency treatment shows that radiofrequency needling was more effective on acne scars, significantly decreased sebum production, and showed higher expressions in regenerating pathways and reduced inflammation markers [12]. Treatment with ultrasound (HIFU, microfocused ultrasound) is based on sound waves that generate heat and mechanical effects in the tissue. The effect is described as tightening/lifting [13, 14] and stimulation of collagen/elastin production [15, 16].

Unlike thermal procedures, mechanical procedures such as microneedling, microdermabrasion, or microvibration treatments involve significantly lower risks, downtime (period of clinical and social transient limitations following treatment), regulatory hurdles, and costs for consumers and practitioners, and have enjoyed increasing popularity in recent years. In microneedling, fine needles create micro‐injuries in the uppermost layers of the skin, thereby activating repair and regeneration processes [17, 18]. Dermabrasion/microdermabrasion is based on the mechanical removal of the uppermost layers of skin (e.g., with crystals or diamond attachments) and is known for skin renewal and the reduction of scars and pigment spots [19]. Meanwhile, vibration therapy/microvibration is based on mechanical stimulation of the skin and subcutaneous tissue, leading to improved blood circulation, lymphatic drainage, skin rejuvenation, and tightening [20, 21].

While there are already numerous literature reviews and comparisons of energy‐based procedures and their mechanisms of action are well known [22, 23], the effects of mechanical device treatments on the skin remain less well understood. Key questions include how different mechanical device treatments affect the user's skin on a clinical and molecular level, which signaling pathways they activate, and which active ingredients can be used to synergistically support regeneration processes at what point in time after the procedure. Only a systematic understanding and comparison of the effects and regeneration phases of the individual treatments enables the tailored use of each procedure according to individual needs, improved results, and increased customer satisfaction—this is what the present review aims to provide.

## 2 Materials and Methods

A systematic and reproducible literature survey was conducted for this overview using the databases Google Scholar and PubMed between September and December 2025. In the first phase, the search focused on studies, reviews, and experimental work on thermal, mechanical, and light‐based skin treatments using the keywords “laser skin therapy”, “fractional”, “ablative”, “Radiofrequency”, “RF‐Needling”, “LED”, “photobiomodulation”, “broad spectrum light therapy”, “ultrasound”. In the second phase, it focused on the three specific mechanical procedures. The search strategy included a combination of relevant keywords related to treatment, such as “microneedling”, “microdermabrasion”, “microvibration” and “device”, furthermore skin reactions, such as “inflammation”, “protein”, “collagen”, “elastin”, and functions such as “pathway”, “response”, “expression”, as well as combinations with active ingredients such as “actives”, “ingredients”, “cosmetics”, or “cosmeceuticals.” Included were publications in English since the year 2000, referred to in vitro and in vivo/ex vivo human or animal experimental models and reported biophysical, molecular, or clinical data. The identified scientific articles were first screened by title and abstract, then reviewed for relevance, methodological quality, and scientific significance after full‐text analysis. Only studies with an appropriate control group were included, while non‐placebo‐controlled studies and those with identifiable conflicts of interest were excluded, ensuring a transparent and replicable search strategy. Supplementary literature from the reference lists of relevant articles was also considered to ensure a comprehensive and up‐to‐date basis for the analysis.

The abbreviations used in the Results and Discussion sections are explained in detail in Table S1 as a glossary.

## 3 Results

### 3.1 Function as Well as Clinical, Structural, and Molecular Effects of Mechanical Treatments

#### 3.1.1 Microneedling

**FIGURE 1:**
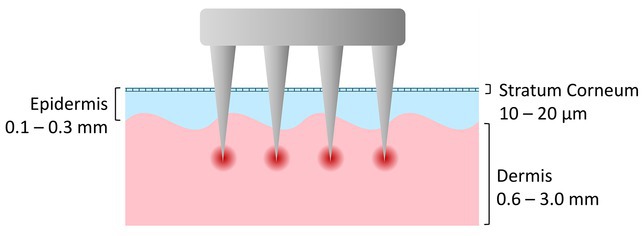
Schematic representation of the microneedling treatment process in an illustrated cross‐section of skin. The microneedles penetrate the epidermis and reach the upper dermis, creating controlled micro‐injuries that induce a local wound healing response with activation of fibroblasts, collagen formation, and increased absorption of topical active ingredients (Graphic created by A.S., 2025).

Microneedling induces localized wound healing through controlled, mechanical micro‐injuries to the epidermis and upper dermis (typically 0.5–2.5 mm needle length). Practitioners distinguish between cosmetic microneedling (up to a maximum penetration depth of 0.5 mm on the face) and medical needling (up to a penetration depth of 2 mm) reaching the dermis layer. The needles are applicated on the one hand via derma rollers, in which radially arranged needles roll over the skin, and derma pens on the other hand, where a motor pierces the needles vertically into the skin at a set frequency. Patches with dissolvable microneedles for improved release of active ingredients are not covered in this review. Depending on the manufacturer, the insertion depths are set or adjustable between 0.3–2 mm and, in the case of electrical devices, at frequencies between 60 and 120 pulses per second—both customizable to user preferences. While the needle spacing on dermarollers is fixed on a drum‐shaped cylinder, with dermapens it can be selected for each treatment according to the individual needs of the patient—sterile cartridges with different needle numbers and shapes are available. With both devices, treatments on the face are performed in special patterns, depending on the addressed skin concern and area, with the needle depth adjusted to the individual's pain tolerance and the current condition of their skin. One treatment session lasts from a few minutes to half an hour, depending on the intensity and area treated [17, 18, 24]. Figure 1 provides a schematic representation of the microneedling procedure.

The **clinically** observed impact is characterized by a temporary erythema and swelling reaction on the treated skin areas, accompanied by slight pinpoint bleeding and minimal pain. These acute reactions usually subside within a few hours to days, while the skin structure visibly improves over the following days to weeks as collagen remodeling and epidermal regeneration progress. It is mainly applied to scars (caused by acne or injury), fine wrinkles, sagging skin, stretch marks, large pores, or uneven skin, and achieves a refining effect [25]. A half‐face study investigating the effect of dermapens and dermarollers on scars showed that, although both sides underwent significant changes, in the end, both methods led to the same results. However, patients treated with dermarollers reported more pain, erythema, and bleeding [26].

At the **structural level**, we focus the effect on cells, the cell interior, and the extracellular space, as well as matrix proteins. A literature review on the mechanism of action of microneedling described a change in electrical potential due to puncture (up to 3 mm depth with a 1.5 mm puncture), followed by a change in the charge of the intercellular space and the formation of an electromagnetic field (200 punctures per cm^2^ are necessary for a measurable effect). As a result, scar tissue was remodeled and new capillaries and fibroblasts were incorporated [27].

The effect at the **molecular level** can be determined by histological immunological examination of tissue (in vitro or ex vivo). In a treatment of human skin models with a dermaroller at a depth of 1 mm, upregulation of genes responsible for wound healing (COL3A1, COL8A1, TIMP3), cell proliferation (KRT13, IGF1), the immune system (CCL11), and HSPB6 was observed. Cytokines (IL‐1α, IL‐1β, IL‐24, IL‐36γ, IL‐36RN) and antimicrobial peptides were downregulated (S100A7A, DEFB4) [28]. On the other hand, matrix metalloproteinases (MMPs) were upregulated, resulting in scar tissue degradation [26].

#### 3.1.2 Microdermabrasion

**FIGURE 2:**
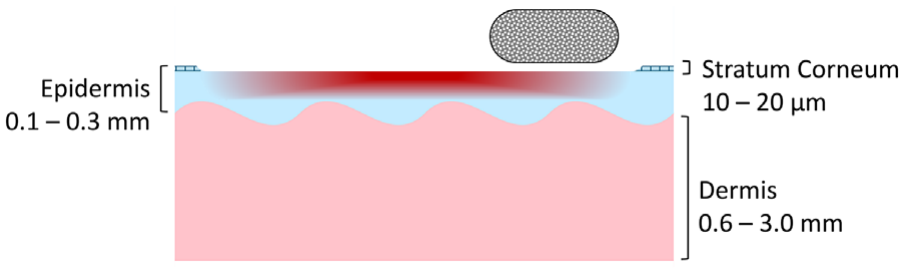
Schematic representation of the microdermabrasion procedure in an illustrated cross‐section of skin. Mechanical removal of the uppermost layer of the epidermis using crystalline particles or a diamond attachment removes dead skin cells, smooths the skin's surface, and stimulates regeneration of the epidermis, thereby promoting the absorption of topical active ingredients and collagen synthesis in the upper dermis (Graphic created by A.S., 2025).

In microdermabrasion, fine crystals are directed over the skin using a handpiece and a vacuum system, removing and suctioning away excess skin cells from the uppermost layer of the epidermis. We distinguish between crystal‐based systems, which most commonly use aluminum oxide, sodium bicarbonate, or ceramic crystals, and systems with abrasive attachments, e.g., made of diamond particles. Water jet‐based dermabrasion is not discussed in this review. The intensity of the treatment can be controlled by the vacuum strength, the speed of the handpiece, the number of overlapping areas and the application patterns. The treatment lasts between 10 and 30 min, depending on the intensity and the area treated [19, 29]. Figure 2 illustrates the microdermabrasion treatment schematically.

**Clinically** and structurally, the treatment of individuals with microdermabrasion over 12–14 weeks evaluated by self‐assessment and histology showed improvement in roughness, pigmentation, epidermal hyperplasia and a partial reduction of acne scars [30]. While this study also found an increase in elastin production, another study only observed an increase in collagen fiber density and no change in elastin after 8 weeks. Significant changes in epidermal thickness, reduction in pigmentation, and improvement in melanosome distribution were also observed here, but overall only moderate clinical changes occured [31]. In a randomized half‐face test investigating the influence of the treatment intensity of microdermabrasion in 6 sessions at two‐week intervals, no difference was found between skin areas treated with two versus three consecutive abrasion passes per session in terms of sebum content immediately after treatment. With triple abrasion, sebum content was higher 1 week after the last treatment in the series, while the pH value was higher at all measurement times with double abrasion. This indicates an influence on the skin barrier [32]. Furthermore, research on guinea pigs suggests that the skin barrier was regenerated after 12 h, the stratum corneum was restored after 24 h, and the skin remained more permeable to active ingredients for the first 12 h. However, it is assumed that recovery periods are longer in humans [33]. A histological evaluation of people treated with six sessions of aluminum oxide microdermabrasion compared to untreated areas showed that microdermabrasion led to thickening of the dermis and epidermis, skin smoothing, increased blood flow, and new collagen and elastin formation [34]. In a direct comparison with a salicylic acid peel treatment, people who underwent microdermabrasion six times at weekly intervals showed thickening of the epidermis and an increase in collagen and elastin, with all effects being stronger with salicylic acid [35].

At the **molecular** level, treatment of humans with dermabrasion with and without crystals showed a significant increase in AP‐1, IL‐1b, TNF‐a, MMP‐1, and MMP‐3, demonstrating that abrasion is necessary to activate signaling pathways [36]. Meanwhile, another study on the treatment of people with UV‐damaged skin using aggressive microdermabrasion with diamond crystals revealed that wound healing signaling pathways were activated (AP‐1, MMPs, HSP47, prolyl 4‐hydroxylase, procollagen types I and III). This remodeling could not be achieved with a less abrasive handpiece [37]. Even after a single microdermabrasion treatment, an increase in transcription factors, cytokines, and MMP was observed immediately after treatment, with isolated increase in procollagen type I messenger RNA after 14 days [38]. For more in‐depth insights, we refer to literature reviews on this subject [39, 40].

#### 3.1.3 Microvibration

**FIGURE 3:**
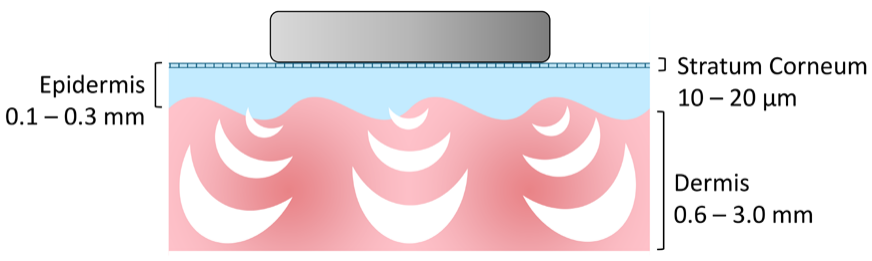
Schematic representation of the microvibration procedure in an illustrated cross‐section of skin. The vibrations enhance microcirculation, improve lymphatic drainage, and promote cellular metabolism by delivering gentle mechanical stress to fibroblasts and keratinocytes, thereby promoting the release of growth factors and modulation of inflammatory mediators, leading to improved tissue oxygenation and accelerated repair processes (Graphic created by A.S., 2025).

Microvibration therapy is a non‐invasive biophysical treatment that uses low‐frequency mechanical oscillations to stimulate skin and underlying tissues. The vibrations enhance microcirculation, improve lymphatic drainage, and promote cellular metabolism by delivering gentle mechanical stress to fibroblasts and keratinocytes. This mechanical stimulation triggers the release of growth factors and modulates inflammatory mediators, leading to improved tissue oxygenation and accelerated repair processes, as shown in Figure 3. In practice, the treatment is performed using a handheld device equipped with vibrating heads that move across the skin in circular or linear motions for several minutes per area, typically without causing discomfort or downtime [20, 21].

According to the authors' current knowledge, the studies on the **clinical effect** of microvibration treatment on facial skin are still underrepresented. However, after 15 vibration treatments of buttocks and thighs lasting 30 or 60 min each, significant changes in skin temperature were observed after each treatment and a clear reduction in the degree of cellulite after the treatment series, with 60 min showing the strongest cellulite‐improving effects [41]. This effect is supported by two further investigations [42, 43]. A similar study observed improved skin hydration and pH and reduced TEWL after treatment—however, no long‐term effect was described [44]. Although the studies were performed on the body and their transferability to the face still requires investigation, these are clear indications of a clinical effect.

The influence of mechanical vibration frequencies on **structural proteins** was investigated, revealing significant increases in collagen IV and tropoelastin under 90 and 120 Hz application, while 60, 90, and 120 Hz had an increasing effect on collagen VII, laminin 5, perlecan, fibronectin, and procollagen I [45]. Another study of different vibration frequencies between 65 to 85 Hz on skin models and humans showed, in comparison with untreated skin, an expression of decorin, fibrillin, tropoelastin, and procollagen I, strongest at 75 Hz [46]. Dermal fibroblasts are particularly sensitive to mechanical nano‐stimulation leading to activation of intracellular signaling pathways that regulate fibroblast proliferation, cytoskeletal remodeling, and extracellular matrix synthesis (mechanotransduction). However, the translatability of the in vitro> results to practice remains uncertain [47]. Overall, mechanical stimulation is known to influence fibroblast activity and leads to increased synthesis of extracellular matrix components such as collagen, elastin, and fibronectin by regulating the activity of MMPs and their inhibitors (TIMPs) [21]. Regarding the **inflammatory response**, a study of the effect of vibration frequencies at 45 Hz on keratinocytes in skin models showed restructuring in the cytoskeleton, as well as activation of the ERK1/2 signaling pathway and activation on gene expression level (HB‐EGF and EGFR). It follows that vibrations can accelerate wound healing [48]. A literature review has already described how vibrations increase the expression of CYP1B1 and stimulate ROS in low concentrations, which in turn express TNFs, TGF‐b1, FGF, and VEGF. The mechanical influence on LINC and mitochondria as well as piezoelectric and thermal factors were discussed [20].

Before discussing the distinctions between these modalities, Table 1 provides a concise overview of their mechanisms of action, clinical indications, and indicative evidence levels.

**TABLE 1:** Summary of the mechanisms of action, primary clinical indications, and indicative evidence levels of mechanical skin‐treatment modalities (microneedling, microdermabrasion, and microvibration).

Modality	Mechanism of action	Clinical indication	Evidence level	Literature
Microneedling	Controlled deep needle insertion to epidermis/upper dermis induces wound healing cascade and vascular formation.	Scars (acne/trauma), fine wrinkles, skin laxity, stretch marks, enlarged pores, uneven skin texture.	Strong evidence: multiple small clinical studies (incl. half‐face and combination studies), one systematic review, numerous narrative reviews, and strong mechanistic evidence (3D skin models, gene expression studies).	[17, 18, 24, 25, 26, 27, 28]
Microdermabrasion	Superficial mechanical exfoliation of the stratum corneum activates wound‐healing pathways and stimulates epidermal turnover.	Skin roughness, pigmentation disorders, acne scars, photoaging, uneven texture.	Strong evidence: small prospective clinical studies with histology (*n* ≈6–30), one randomized half‐face trial, multiple mechanistic human studies (gene expression, MMPs), in vitro penetration studies, and animal barrier‐recovery models.	[19, 29, 30, 31, 32, 33, 34, 35, 36, 37, 38, 39, 40]
Microvibration	Low‐frequency mechanical oscillations induce mechanotransduction and increase microcirculation, lymphatic drainage, and cellular metabolism.	Cellulite, skin laxity, impaired circulation, hydration/barrier dysfunction (transferability to facial indications not fully established).	Moderate evidence: mostly small human clinical studies (split‐face, body applications, 15–60 min sessions, *n* ≈10–30), ex vivo and in vitro mechanistic studies on fibroblasts and skin models.	[20, 21, 41, 42, 43, 44, 45, 46, 47, 48]

### 3.2 Repair Process and Methodological Differences

**FIGURE 4:**
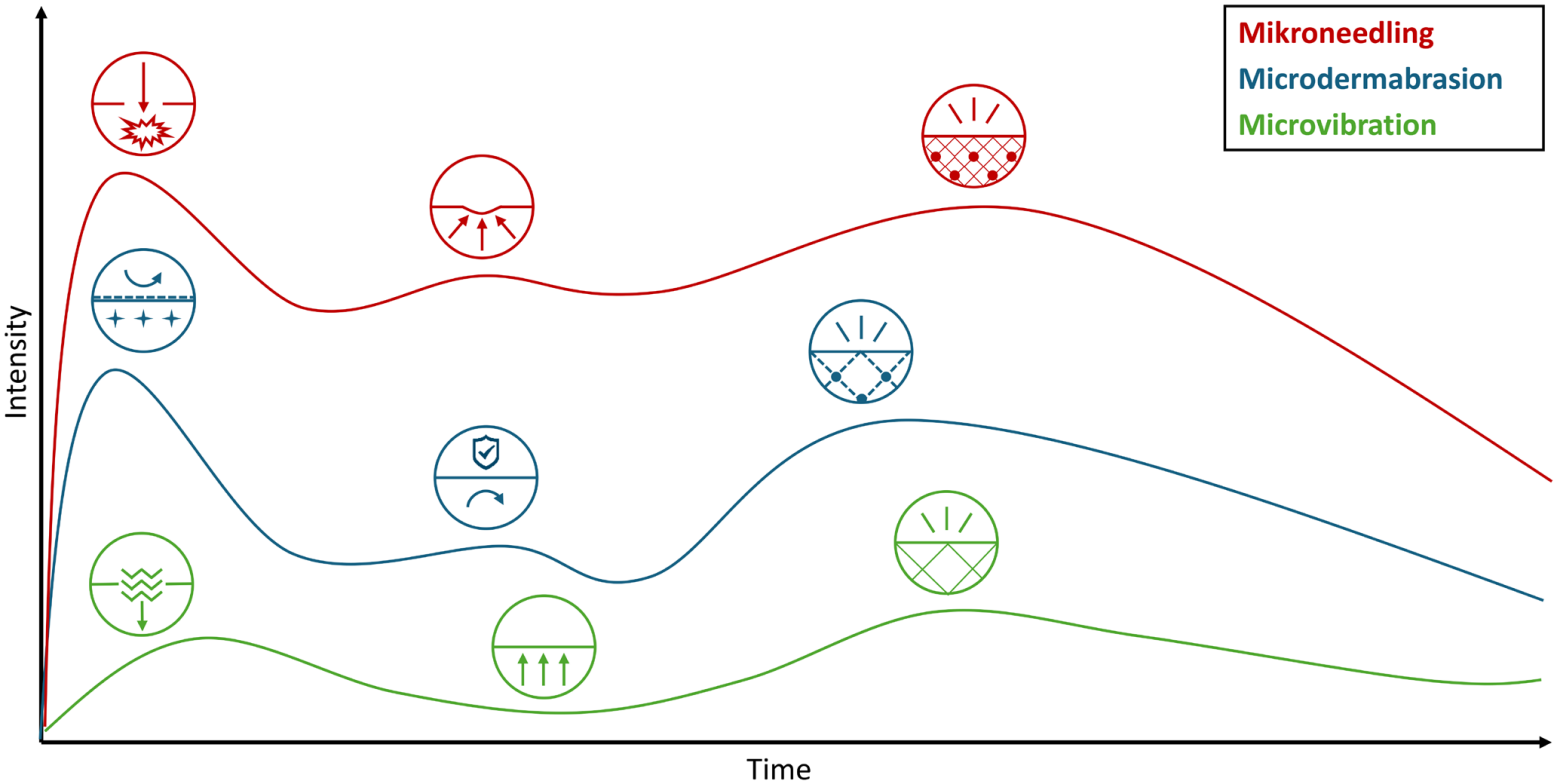
Proposed tissue reaction over post‐treatment and recovery phases after microneedling, microdermabrasion, and microvibration treatment. All three phases (inflammation/exudation, proliferation/granulation, remodeling/epithelialization) are most strongly affected by microneedling, which also has the longest‐lasting effect, while microdermabrasion also causes inflammation and remodeling, whereas microvibration only shows activities relevant to remodeling. While the inflammatory phase in microneedling and microdermabrasion is caused by puncture/abrasion and recovers in the following period, microvibration activates the tissue through the impulses. In the remodeling phase, collagen (cross‐linked lines) and elastin (spheres) are formed at different intensities in all treatments. (Graphic created by A.S., 2025).

It is commonly accepted that the skin heals and recovers superficial wounds in the following phases: (a) hemostasis (stopping bleeding), (b) inflammation/exudation (removal of foreign material/breakdown of damaged cells in the epidermis and upper dermis), (c) proliferation/granulation (restoration through re‐epithelialization in the epidermis and granulation tissue formation in the superficial dermis), and (d) remodeling/epithelialization (reconstruction of the newly formed tissue in the dermis to strengthen the skin) [49]. Since microneedling perforates several layers of the skin, it should show the strongest reaction in all phases [17, 18, 24, 25, 26, 27, 28], whereas with microdermabrasion, the proliferation phase should not be as intense, as no new cells need to be formed in the epidermis and dermis (only re‐epithelialization) [19, 29, 30, 31, 32, 33, 34, 35, 36, 37, 38, 39, 40]. While dermabrasion and microneedling are often categorized as mechanical interventions, both modalities intentionally induce tissue injury, thereby activating wound‐healing cascades and inflammation‐driven repair processes. In contrast, microvibration represents a non‐injurious mechanical stimulus whose biological effects are primarily mediated through mechanotransduction rather than tissue damage. Mechanotransduction refers to the ability of cells to sense and convert mechanical cues into biochemical signals, ultimately influencing gene expression and cellular function. Although it does not damage the skin at all, it still triggers slight oxidative stress and remodeling reaction [20, 21, 41, 42, 43, 44, 45, 46, 47, 48]. The graph in Figure 4 shows a comparison of the recovery and renewing phases for the three treatments.

**FIGURE 5:**
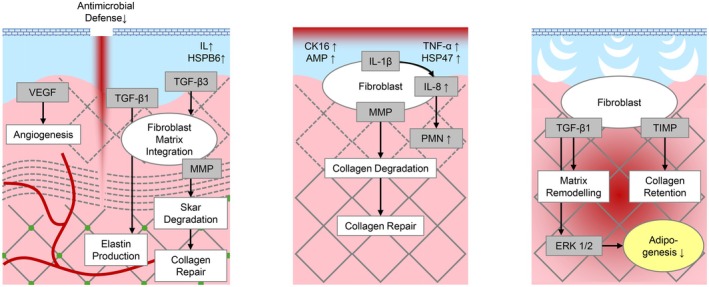
Molecular tissue reaction after microneedling, microdermabrasion, and microvibration treatment. All methods contribute to collagen formation in similar yet different ways (Graphic created by A.S., 2025, inspired by Liebl & Kloth (2013) [27], Karimipour et al. (2009) [37], and Kołodziejczak et al. (2025) [21]).

Table 2 shows a direct comparison of the effects of microneedling, microdermabrasion, and microvibration in terms of clinical effects, structural changes, molecular markers in the skin, and the suitability of the methods for different skin conditions. Figure 5 simplifies the recovery process and its effects on the respective skin layers.

**TABLE 2:** Comparison of the treatment effects of microneedling, microdermabrasion, and microvibration [17, 18, 19, 20, 21, 24, 25, 26, 27, 28, 29, 30, 31, 32, 33, 34, 35, 36, 37, 38, 39, 40, 41, 42, 43, 44, 45, 46, 47, 48]: Clinical, structural, and molecular mechanisms.

Parameter	Microneedling	Microdermabrasion	Microvibration
Mechanism of action	Wound healing/skin repair after needle puncture	Epidermal recovery after removal of the surface epidermal layer	Skin modulation after mechanical stimulation of skin and underlying tissue
Clinical			
Acute Tissue Reaction	Erythema, punctate hemorrhage (most intense within the first hour, lasts for a few hours)	Erythema and sebum changes depending on intensity	Local hyperemia and temperature increase
Healing Duration	Epidermis within days, structurally within weeks to months	Stratum corneum > 24 h, structurally within weeks	No healing required superficially, structurally within weeks
Visual Outcome	Reduction of scars, hyperpigmentation, fine lines, and wrinkles	Improvement in skin roughness, cornification/thickness, pigmentation, deep cleaning effects, partial reduction of acne scars	Improved smoothness, firmness and hydration; reduction of cellulite and edema
Structural			
Skin Barrier Impact	Remains largely intact	Reduced, more permeable in the first 12 h	Remains intact
Tissue Changes	Scar tissue remodeling; integration of new capillaries and fibroblasts; electrical potential change and EM field	Epidermal hyperplasia, thickening of the dermis and epidermis, skin smoothing, improved melanosome distribution, increased blood circulation	Increased fibroblast activity and synthesis of Collagen, Elastin, Fibronectin; improved microcirculation; cytoskeletal restructuring
Matrix Proteins	Elastin, Collagen ↑	Collagen ↑, Elastin ↑ only in longer treatment periods, Procollagen I & III ↑	Collagen, Tropoelastin, Laminin 5, Perlecan, Fibronectin, Procollagen I ↑
Molecular			
Cell Proliferation	COL3A1, COL8A1, KRT13, IGF1, NF‐kB, TGF‐β ↑	AP‐1 & NF‐kB ↑	COL1A1, ELN & FN1 ↑; ERK1/2 pathway activation; HB‐EGF, EGFR ↑ TGF‐β1, FGF, VEGF ↑
Enzymes	MMP, TIMP3 ↑	MMP‐1, MMP‐3 &‐9 ↑	MMP ↓, TIMP1, TIMP3 ↑
Stress Response	HSPB6 ↑	HSP47 & prolyl‐4‐hydroxylase ↑	ROS ↑
Inflammation	CCL11 ↑, IL‐1α, IL‐1β, IL‐8, IL‐24, IL‐36γ & IL‐36RN ↓	IL‐1β, TNF‐α ↑	Not described
Antimicrobial Defense	S100A7A & DEFB4 ↓	Not described	Not described

### 3.3 Usage of Active Ingredients During Treatments With Mechanical Devices

Some publications describe increased effectiveness of active ingredients and cosmetic products after **microneedling** [50, 51]. For example, a study on the effect of vitamins A and C in combination with quadruple microneedling at a depth of 1 mm in rats indicated an increase in epidermal thickness and dermal structure that was stronger in the presence of the active ingredients than in their absence. Gene expression analysis found an increase in the growth factors TGF‐β1‐3, FGF, EGF, VEGF, TNF‐α, and collagen I, while collagen III decreased [52]. Treatment of humans with microneedling (Dermaroller 1.5 mm) and a serum containing unsaturated fatty acids, niacinamide, and plant extracts (active serum) compared to a hyaluronic acid serum (control) in the hemifacial test showed significantly lower redness after 7 days, significantly higher brightness, and more even texture at all measurement points on the side with the active serum [53]. Although there was no indication regarding randomization of the serum side and typical facial asymmetry effects cannot be completely ruled out, both studies clearly show the supporting effect of cosmetic ingredients on tissue repair processes after the procedure. This is due to increased penetration of the active ingredients and thus improved efficacy, as has already been described for minoxidil [54], glycolic acid [55], and other cosmeceuticals [56]. As a result, numerous substances that are already approved for use in cosmetic applications also appear to be effective in combination with microneedling. Nevertheless, clear evidence of concrete penetration and depth, as well as the connections with the applied formulation and matrix under microneedling, are still lacking.

In addition to ingredients, microneedling treatments with the patient's own blood are becoming increasingly popular, due to the growth factors contained therein [57]. Treatment with platelet‐rich plasma (PRP) induced significantly more collagen, elastin, epidermal proliferation, and caspase‐3 protein expression compared to microneedling without PRP [58]. Similar findings have been reported regarding the use of platelet‐rich fibrin (PRF) with microneedling [59]. Inspiration from these approaches to cosmetic and dermatological active ingredients could increase product efficacy and treatment effectiveness. One study approaching this idea examined repeated microneedling twice a week at home with a depth of 0.2 mm over a period of 3 months using a growth factor serum, followed by the application of a regenerative serum twice a day. Both products contained bFGF, aFGF, IGF‐2, IGF‐1, KGF‐2, TGF‐β3, PDGF‐A, KGF‐1, HGF, EGF, and the regenerative serum also contained isoflavones, peptides, antioxidants, moisturizers, DNA repair enzymes, ultrasomes, and retinol. The result was a significant improvement in skin texture, wrinkles, redness, pigmentation spots, and satisfaction [60]. Despite the absence of an untreated control group, the study demonstrated the practical feasibility of a skin care serum containing growth factors to support skin regeneration. Growth factors are therefore not only a relevant in situ mechanism but can also serve as a topical active ingredient for more effective treatments.

In case of **microdermabrasion**, studies have investigated the skin penetration of active ingredients as well. In vitro, the penetration of niacinamide improved, while the treatment had no effect on the action of retinol. The authors concluded that penetration depends on lipophilicity [61]. A study on guinea pigs investigating the efficacy of active ingredients for repigmentation showed that neither dermabrasion nor the active ingredient alone could repigment the skin. A synergistic effect occurs only in combination of both, active ingredient plus dermabrasion [62]. In cases of skin changes after burns, scars, and acne, a combination of microdermabrasion with an active ingredient showed significant improvements [63]. While microneedling, microdermabrasion, and tapestripping increased the absorption of active ingredients, massage and ultrasound reduced it [64]. A systematic literature review also showed that skin penetration of active ingredients depended on their lipophilicity, time of application, and treatment. In this case, however, massage increased the absorption of active ingredients [65]. Overall, these findings suggest that the enhancement of active ingredient absorption is highly context‐dependent, influenced not only by the type of mechanical intervention but also by the physicochemical properties of the compounds and the timing of their application.

**Microvibrations** increase the absorption of active ingredients, as studies have already shown. The highest transdermal absorption of nanoactive ingredients in pig skin was achieved at 4.2 Hz, the lowest at 100 Hz [66]. Similar results were observed in a study on the human skin penetration of soluble active ingredient particles with simultaneous application of vibrations, whereby the vibrations resulted in better penetration depth of the particles and distribution of the active ingredients [67]. These findings are supported by the observation that an 8‐week massage treatment with the application of a cream showed a stronger anti‐aging effect than the use of the cream alone [46]. Additionally, in a randomized half‐face study, the effect of vibration‐assisted cleansing compared to conventional skin cleansing was investigated, whereby the vibrations led to significantly smaller pore size [68]. These findings indicate that microvibrations do not physically breach the skin barrier, but they enhance the absorption of active ingredients by loosening the intercellular structure.

## 4 Discussion

### 4.1 Comparison of the Focused Mechanical Treatments

Tables 1 and 2 clearly show that the clinical, structural, and molecular processes of the three focused treatments (microneedling, microdermabrasion, and microvibration) differ, but also overlap and share similarities. A fundamental difference lies in terms of the underlying technology and the associated mechanism of action: puncture vs. removal vs. stimulation. Given this background, the different processes and effects in the individual skin layers (surface, epidermis, dermis, subcutis) can be deduced. Clinically described, the acute tissue reaction of microneedling and microdermabrasion shows similarities, which are also reflected in the comparable outcome (improved skin texture, pigmentation, and scars), whereas microvibration has particularly positive effects in terms of roughness, firmness, and moisture balance. Looking at the expression of matrix proteins, it becomes clear that all three methods have a positive effect on the total collagen and elastin content, although the types of increased collagen and elastin fibers appear to differ slightly and procollagen increase is only shown for microneedling and microvibration. After all mechanical treatments, cell proliferation is increased, whereas molecular upregulation and expression differ. However, it is described that TGF‐β and NF‐κB play a key role after mechanical treatments. This also applies to the interaction and balance between the MMP and TIMP up‐ and downregulation, which is also influenced by all three analyzed mechanical treatments. Interestingly, reliable data on growth factor upregulation is currently only available for microvibration, whereby the formation appears to be triggered by slightly increased ROS levels [20]. Analyzing the inflammatory response shows that microneedling and microdermabrasion lead to complex cascades and processes of several cytokines or their antagonists. Inflammatory response has not yet been reliably examined for microvibration, and antimicrobial defense is only described for microneedling, in terms of downregulation of S100A7A and DEFB4 [17, 18, 19, 20, 21, 24, 25, 26, 27, 28, 29, 30, 31, 32, 33, 34, 35, 36, 37, 38, 39, 40, 41, 42, 43, 44, 45, 46, 47, 48].

Taking all clinical, structural, and molecular processes and mechanisms of actions into account (Tables 1 and 2, Figure 5), it becomes clear that microneedling, microdermabrasion, and microvibration are close in terms of effectiveness, but also regarding the clinical aesthetic outcome. However, each mechanical treatment method has its own characteristics, depth, and peculiarities and should therefore be used specifically for different consumer needs and aesthetic targets. Therefore, the focus of microneedling is on positively influencing skin texture, as well as reducing irregularities and unevenness (scars, pores, pigments) based on strong and deep remodeling effects with long‐lasting structural improvement. In comparison, microdermabrasion mainly improves skin surface, cornification, thickness, removes impurities, reduces superficial discoloration and irregularities, resulting in instant even, clear, and smooth skin. Instead, microvibration shows strong effects on deeper tissue by improving microcirculation, cytoskeletal restructuring to stimulate cell activity and metabolism. In this context, microvibration may act as a controlled mechanical signal that activates mechanotransduction pathways without causing micro‐injury. This distinguishes microvibration from ablative or minimally invasive rejuvenation techniques and supports its potential role as a novel, non‐damaging modality for skin rejuvenation. By selectively engaging mechanoresponsive pathways in fibroblasts, microvibration may promote regenerative cellular functions while minimizing inflammatory responses associated with tissue injury [17, 18, 19, 20, 21, 24, 25, 26, 27, 28, 29, 30, 31, 32, 33, 34, 35, 36, 37, 38, 39, 40, 41, 42, 43, 44, 45, 46, 47, 48].

To conclude, microneedling mainly provides deep remodeling effects and structural improvement, microdermabrasion delivers superficial refinement, and microvibration is mainly effective for general activating and stimulating the skin in different layers. Aside from the specific and targeted application of one single mechanical treatment, it could be therefore very effective to combine these methods, consecutively or alternately. Combinations can improve the desired and targeted result depending on the existing skin type and condition, such as the very effective alternating application of microneedling and microdermabrasion [24, 29].

### 4.2 Comparison of Mechanical and Thermal Treatments

Although mechanical procedures such as microneedling, microdermabrasion, and microvibration treatments work without direct heat exposure, they show many parallels to thermal procedures (see Table 1) [1, 2, 3, 4, 5, 6, 7, 8, 9, 10, 11, 12, 13, 14, 15, 16]. Heat exposure in the range of approximately 42°C–43°C induces a defined cellular stress response characterized by temperature‐dependent upregulation of central heat shock proteins such as HSP70 (rapid and pronounced activation as a primary marker of protein denaturation), HSP47 (increased expression above 42°C in connection with collagen synthesis and long‐term wound healing), and HSP27 (rapid increase in mRNA from around 42°C), and at the same time the activation of inflammatory signaling pathways such as NF‐κB and MAPK with increased expression of TNF‐α, IL‐6, and IL‐1β, a shift in the redox environment via Nrf2‐ and PI3K‐Akt‐dependent mechanisms, and modulation of apoptotic processes via Bcl‐2, Bax, and caspases, and the involvement of other regulatory networks such as MAPK, Wnt/β‐catenin, and TGF‐β [22]. A recent half‐side test with fractional radiofrequency needling in direct comparison with microneedling found that the number of HSP47+ fibroblasts increased significantly after radiofrequency needling, as did procollagen I, hydration, dermal collagen density, and elastin [69]. However, mechanical procedures such as microneedling do not cause heat necrosis, and wound healing is characterized by non‐inflammatory processes based solely on TGF‐β, with the skin barrier remaining largely intact [17]. Although thermal methods appear to be more effective, the lower risks of mechanical treatments might benefit from lower hesitation among consumers and practitioners while still being convincingly effective.

### 4.3 Synergy With Active Ingredients

Given the well‐characterized tissue repair and recovery phases (Figure 4) and the effects of mechanical treatments on the skin at clinical, structural, and molecular levels (Table 1), it remains to determine what medical and cosmetic products are required to optimally support regeneration during and after treatment, supporting and nourishing the skin with the appropriate “building blocks” at the right time. Immediately after treatment, soothing and barrier‐supporting ingredients (antioxidants, niacinamide) might accelerate healing and suppress excessive inflammatory responses and oxidative stress [61]. Especially in the initial phase after microneedling, when antimicrobial defense is reduced and the skin microbiome is altered [28], ingredients and products with gentle and microbiome‐friendly properties are advisable. An effective preservation is absolutely mandatory under these application scenarios. Growth factors could be helpful during the proliferation phase. When applying structure‐building ingredients (retinoids, vitamin C derivatives), it might be worth waiting until the remodeling phase to promote collagen production without risking adverse effects, such as sensitivity [70].

Although our results indicate that mechanical treatments are able to improve skin penetration of active ingredients, a comparison between thermal and mechanical treatments should also be considered, since different procedures affect the skin layers differently. For example, a study comparing lasers treatment vs. microdermabrasion showed that although Erb:YAG improved transdermal absorption the most (1000–4000 μg/cm^2^ after 12 h), followed by CO_2_ laser (50–1000 μg/cm^2^ after 12 h), microdermabrasion was still 20 times higher than untreated skin at approximately 200–400 μg/cm^2^ after 12 h [70]. Therefore, the choice of active ingredients and their concentration should be adapted to the treatment, since the treatment itself might alter the penetration, efficacy, and compatibility of the active ingredients, directly impacting clinical outcomes, side effects, and individual treatment decisions.

Although the literature confirms the safety of microneedling (any adverse effects would be mild and would quickly subside), however, particular caution is advised in cases of darker skin types, metal allergies, pre‐existing infections, and products that are not approved for subcutaneous application [71]. One critical literature review on the inflammatory response to microneedling suggests that repeated microneedling is associated with systemic and dermatological inflammatory responses and that active ingredients with human donor bone marrow stem cell secretomes might enhance this [72]. Although the appropriate frequency and ingredients were not specifically mentioned, this supports the need that medical and cosmetic products should be assessed and approved for subcutaneous application by microneedling, as they meet different demands than products for intact skin barriers. Approved “skin boosters” for injection [73] may provide useful guidance in this regard.

Additionally, since several treatments compromise the skin barrier to some degree, products used during and after treatment should be microbiologically safe, for instance through skin‐friendly preservation and contamination‐proof packaging (disposable packaging or airless pump dispensers instead of pipette droppers) are preferable Especially while the expression of antimicrobial factors in the skin is reduced [28], the product should be designed to compensate for this. In addition, greater focus should be placed on the influence of those products with regard to the skin microbiome in the future, as it is already known that the skin microbiome has an important effect on the severity and regulation of inflammation [74].

### 4.4 Potential for Further Research

To the best of the authors' knowledge, there is currently no study that compares all three mechanical treatment types in vivo. Although this literature review is well suited to structuring previous results, the heterogeneity of the study designs brings limitations in comparison. On the one hand, the current level of evidence for microvibration treatments remains limited and would benefit substantially from studies of similar methodological rigor to those already published for microneedling and microdermabrasion. On the other hand, a study with uniform conditions for all three mechanical treatments in comparison should be conducted in practice and focus on clinical, structural, and molecular mechanisms under the same conditions.

With regard to diversity and inclusion, it would be advisable to conduct additional efficacy studies across different skin types (e.g., photo‐types, skin conditions, genders, etc.) and at various stages of life (youth, adulthood, menopause, senior age). At the same time, we are fully aware of the ethical and organizational challenges associated with carrying out such studies as well as with the analysis and interpretation of the resulting data. However, it is well known that different regenerative properties vary depending on skin type and stage of life [75, 76]. A comprehensive understanding of the mechanisms of action and appropriate support with active ingredients is only possible when all specific aspects are considered.

Furthermore, there is a lack of systematic and comparative studies evaluating individual active ingredients or defined combinations of active ingredients in relation to mechanical treatment modalities. Controlled experimental investigations in which specific active ingredients are assessed as single compounds (i.e., in vitro/ex vivo, skin model) would therefore be warranted. Such studies could elucidate regenerative, structural, and inflammatory effects at the molecular and biochemical levels, thereby facilitating the identification of novel dermatocosmetic candidates and improving the understanding of the underlying biological mechanisms.

## 5 Conclusion

The results of this work include similarities, differences, and practical recommendations for skin treatments with microneedling, microdermabrasion, and microvibration. Mechanical treatment modalities vary in their mechanism of action, penetration depth, and biological impact; consequently, their application should be tailored to distinct consumer profiles and defined aesthetic objectives. In summary, microneedling is mainly associated with profound dermal remodeling and long‐term structural enhancement, while microdermabrasion acts chiefly on the epidermal surface to improve skin texture, and microvibration primarily promotes functional activation and stimulation of the skin at various depths. However, further research is needed to evaluate the effects in practice.

Although mechanical procedures are perceived as less intensive than thermal treatments, they activate similar mechanisms in the skin. The phases of this activation should be considered when choosing cosmetic skin care products. Targeted product development offers great synergistic potential for nourishing the skin according to its needs in all healing stages. Our research shows that certain ingredients authorized for both medical and cosmetic use (established actives such as retinoids, ascorbic acid, or niacinamide, but also unsaturated fatty acids, antioxidants, and growth factors) can improve recovery, reduce downtime, and improve the outcome of mechanical aesthetic treatments.

## Author Contributions

J.B. conceptualized the review, supervised the project and provided critical revisions. E.H. reviewed the manuscript, provided internal unpublished literature and critical revisions from a practical perspective. A.S. performed the literature search, data analysis/synthesis, visualisation and drafted the manuscript. All authors contributed to the interpretation of the data, reviewed the final version of the manuscript and approved its submission.

## Funding

This work was supported by Dr. Babor GmbH & Co. KG, Neuenhofstraße 180, 52 078 Aachen, Germany. A.S. received compensation as a Freelance Scientific Consultant for work related to this manuscript.

## Ethics Statement

The authors have nothing to report.

## Conflicts of Interest

The authors declare no conflicts of interest.

## Supporting information


**Table S1:** Glossary of abbreviations.

## Data Availability

Data sharing not applicable to this article as no datasets were generated or analysed during the current study.
